# Mobile App-Delivered Cognitive Behavioral Therapy for Insomnia: Feasibility and Initial Efficacy Among Veterans With Cannabis Use Disorders

**DOI:** 10.2196/resprot.3852

**Published:** 2015-07-17

**Authors:** Kimberly A Babson, Danielle E Ramo, Lisa Baldini, Ryan Vandrey, Marcel O Bonn-Miller

**Affiliations:** ^1^ National Center for PTSD VA Palo Alto Health Care System Menlo Park, CA United States; ^2^ Department of Psychiatry University of California-San Francisco San Francisco, CA United States; ^3^ National Center for PTSD VA Palo Alto Health Care System Menlo Park, CA United States; ^4^ Johns Hopkins University Department of Psychiatry and Behavioral Sciences Johns Hopkins University Baltimore, MD United States; ^5^ National Center for PTSD and Center for Innovation to Implementation VA Palo Alto Health Care System Menlo Park, CA United States; ^6^ Philadelphia VAMC Center of Excellence in Substance Abuse Treatment and Education Philadelphia, PA United States; ^7^ University of Pennsylvania Department of Psychiatry Philadelphia, PA United States

**Keywords:** cannabis, marijuana, sleep, CBT-I, intervention

## Abstract

**Background:**

Cannabis is the most frequently used illicit substance in the United States resulting in high rates of cannabis use disorders. Current treatments for cannabis use are often met with high rates of lapse/relapse, tied to (1) behavioral health factors that impact cannabis use such as poor sleep, and (2) access, stigma, supply, and cost of receiving a substance use intervention.

**Objective:**

This pilot study examined the feasibility, usability, and changes in cannabis use and sleep difficulties following mobile phone–delivered Cognitive Behavioral Therapy for Insomnia (CBT-I) in the context of a cannabis cessation attempt.

**Methods:**

Four male veterans with DSM-5 cannabis use disorder and sleep problems were randomized to receive a 2-week intervention: CBT-I Coach mobile app (n=2) or a placebo control (mood-tracking app) (n=2). Cannabis and sleep measures were assessed pre- and post-treatment. Participants also reported use and helpfulness of each app. Changes in sleep and cannabis use were evaluated for each participant individually.

**Results:**

Both participants receiving CBT-I used the app daily over 2 weeks and found the app user-friendly, helpful, and would use it in the future. In addition, they reported decreased cannabis use and improved sleep efficiency; one also reported increased sleep quality. In contrast, one participant in the control group dropped out of the study, and the other used the app minimally and reported increased sleep quality but also increased cannabis use. The mood app was rated as not helpful, and there was low likelihood of future participation.

**Conclusions:**

This pilot study examined the feasibility and initial patient acceptance of mobile phone delivery of CBT-I for cannabis dependence. Positive ratings of the app and preliminary reports of reductions in cannabis use and improvements in sleep are both encouraging and support additional evaluation of this intervention.

## Introduction

Cannabis is the most frequently used illicit substance in the United States [1], with rates of cannabis use disorders rising among particularly vulnerable populations (eg, veterans [2]). Recently, rates of use and treatment admissions have increased, while public perception of harm associated with cannabis use has decreased. There is clear scientific evidence that long-term, heavy use of cannabis can result in negative health effects including addiction [3]. Although a number of interventions are available for the treatment of cannabis use disorders (eg, contingency management, cognitive behavioral therapy [4]), rates of lapse and relapse, particularly among vulnerable populations, remains remarkably high [5-7].

In light of the relatively poor outcomes associated with standard cannabis use disorder treatments, there has been increasing interest in identifying malleable factors associated with heightened relapse risk among individuals dependent on cannabis, specifically for the purposes of intervention development and refinement [8-12]. Indeed, poor sleep quality has received a considerable amount of recent attention as a malleable factor implicated in the maintenance of, and relapse to, cannabis use [8]. Driven by the observation that cannabis may have acute sleep-promoting effects [13,14] and that individuals dependent on cannabis experience heightened sleep difficulties during abstinence [15,16], recent research has documented that many cannabis users specifically report using cannabis to cope with or “treat” sleep difficulties [17,18].

From these findings, the evaluation of existing sleep treatments as clinical tools to aid in the treatment of cannabis use disorders has been proposed, with the aim of improving sleep and reducing individual “self-medication” with cannabis [8]. Initial work in this domain has focused on pharmacological treatments for sleep. Vandrey et al [19] tested the efficacy of zolpidem for the attenuation of sleep disturbances (ie, sleep efficiency, total sleep time, subjective sleep quality) during a cannabis cessation attempt and found strong evidence for the efficacy of zolpidem in terms of reducing poor sleep during cannabis withdrawal. Vandrey’s study was an important first step in determining the efficacy of sleep treatments for those with a cannabis use disorder; however, there has yet to be an examination of behavioral sleep interventions for individuals attempting cannabis cessation.

Cognitive Behavioral Therapy for Insomnia (CBT-I) is the leading behavioral treatment for sleep disturbances [20,21]. Although there has been some initial investigation of the efficacy and clinical benefit of CBT-I among individuals with alcohol dependence [22,23], there has yet to be an investigation of the use of CBT-I among individuals with a cannabis use disorder.

Given identified barriers to substance use disorder care that include access, stigma, supply, and cost [24-27], a mobile phone app could extend reach and utilization of CBT-I among those with a cannabis use disorder. Compared with traditional care, technology can offer personalized treatment for health risk behaviors such as substance use, while using less counselor time and increasing availability at moments of great need. For example, in a randomized trial of 349 alcohol-dependent patients leaving residential treatment, those who received a mobile phone app intervention for 8 months had fewer risky drinking days at 4 months follow-up than those in a usual care control group (1.39 days vs 2.75 days [28]). In the treatment of cannabis use disorders, one recent study showed that computerized delivery of evidence-based psychosocial therapy (Motivational Interviewing+Cognitive Behavioral Therapy+Contingency Management) was as effective as the same therapy delivered by a counselor [29]. There has been one report of an app to treat cannabis use disorder with promising ratings of ease and overall usefulness from users [30]. However, there has yet to be an evaluation of cannabis use outcomes for those receiving treatment through a mobile phone app.

This pilot study aimed to begin addressing these gaps in the literature by determining the feasibility of providing CBT-I to individuals with a cannabis use disorder engaged in a cessation attempt through a mobile phone app. CBT-I was administered via mobile app instead of in-person sessions as this would allow for greater reach of the intervention (ie, rural areas) and consistent fidelity of the intervention. For the purposes of this feasibility study, we sought to examine mobile app engagement, user-friendliness, and use and future interest in use, in order to provide initial data regarding the feasibility and usability of this technology among veterans with a cannabis use disorder. As a secondary aim, we examined preliminary trends in the impact of app use on cannabis and sleep outcomes. Specifically, we hypothesized that receiving CBT-I through an app, within the context of a self-guided cannabis cessation attempt, would be acceptable to study participants and utilization of the app would correspond with a reduction in cannabis use and an improvement in sleep quality.

## Methods

### Recruitment and Participants

#### Recruitment

In terms of recruitment, 40 individuals contacted the lab expressing interest in the study. Individuals leaving a message were contacted up to four times for the purpose of conducting an initial phone screening. Of the original 40 individuals, 28 veterans who contacted the lab were screened over the phone. Of these, 12 individuals were screened out because they did not report current cannabis use, 6 were not veterans, 5 reported no interest in quitting, and 1 individual was eligible but refused to participate. This resulted in a total sample of 4 individuals.

#### Participants

Study participants (N=4) were adult veterans (18 and over) who reported (1) current sleep problems, defined as a total score of 5 or greater on the Pittsburgh Sleep Quality Index (PSQI) [31], (2) a cannabis use disorder, as defined by the Diagnostic and Statistical Manual of Mental Disorders (DSM), 5th edition (DSM-5) [32], (3) a self-reported interest in making a cessation attempt within the next 30 days, and (4) veteran status. The mean age of the sample was 47 years (SD 16.31; range 27-65 years), and all participants were male. Two participants self-identified as Caucasian, 1 black/non-Hispanic, and 1 black/Hispanic. All met criteria for cannabis use disorder, 1 had posttraumatic stress disorder (PTSD), and 2 had a current alcohol use disorder.

### Measures

The Structured Clinical Interview-Non-Patient Version (SCID I-N/P) for DSM-IV [33] was administered at baseline to determine current Axis I diagnostic status. Diagnosis for substance use disorders was derived from DSM-5 criteria [32], with additional questions added to account for any changes in diagnostic criteria between DSM-IV [34] and DSM-5 (eg, withdrawal for cannabis). Trained research study staff conducted all interviews, with diagnoses discussed with and confirmed by the last author.

A Mobile App Use Measure (MAUM) was developed to gather information about participant use of both the experimental (ie, CBT-I) and control (mood-tracking) apps between baseline and 2-week follow-up, as neither app recorded use data. The MAUM prompted participants to report on (1) frequency of app use, (2) length of use per occasion, (3) components of the app that were used most often, (4) user-friendliness of the app, (5) helpfulness of the app, (6) likelihood of future app use, (7) use of the app for sleep improvement, and (8) use of the app for cannabis cessation. The MAUM was administered at 2-week follow-up.

Sleep quality at baseline and 2-week follow-up was assessed with the 19-item Pittsburgh Sleep Quality Index (PSQI) [31]. Participants indicated their usual bedtime, how long it took them to fall asleep, their usual waking time, and their usual hours of sleep per night, as well as responding to questions such as, “During the past month, how often have you had trouble sleeping because you cannot get to sleep within 30 minutes?” and “During the past month, how often have you taken medicine (prescribed or over the counter) to help you sleep?” on a 4-point Likert-type scale (0=not during the past month, 3=three or more times a week). At baseline, all questions referred to the past month; the questionnaire was modified for follow-up assessment to capture sleep quality only during the time since baseline (ie, 2 weeks). Algorithms were used to generate 7 component scores and a global total score (for scoring algorithms, see Buysee [31]). Components of the PSQI include subjective sleep quality (defined as the overall quality of sleep), sleep latency (amount of time to fall asleep), sleep duration (number of hours of actual sleep per night), sleep efficiency (number of hours of sleep divided by number of hours spent in bed), sleep disturbances (a non-specific index of nighttime disturbances including symptoms of sleep apnea, middle insomnia, disturbing dreams, and physical conditions such as pain), sleep medication (use of prescribed or over-the-counter sleep medication), and daytime dysfunction (trouble staying awake and/or decreased energy during the day). Both component and global scores were calculated. The global score of the PSQI had a high level of internal consistency in this sample (Cronbach alpha=.94 baseline, .81 follow-up).

The Timeline Follow*-*Back Interview (TLFB) [35] was administered at baseline and 2-week follow-up to obtain data on frequency and quantity of cannabis, tobacco, and alcohol use during the 90 days prior to baseline and between baseline and 2-week follow-up appointments. Average cannabis use was calculated as the mean quantity of use across each day of the given assessment period. As quantity of cannabis used on each day was indexed by a graphical depiction of varying quantities that ranged from 0 (lowest amount) to 8 (highest amount [36]), with the possible range for average cannabis use across any given period being 0-8. The TLFB has demonstrated good reliability and validity across cannabis using and veteran samples [37,38]. Due to an initial variation in the protocol, for Participant 1, the Marijuana Smoking History Questionnaire (MSHQ) [36] was used to assess baseline cannabis use. The MSHQ is a self-report instrument that includes items pertaining to cannabis smoking rate (lifetime and past 30 days). Two questions assessing (1) frequency of use within the past 30 days, and (2) quantity of use on each occasion (identical to the graphical depiction used in the TLFB) were multiplied and then divided by the total number of days in the assessment period to determine the participant’s past 30-day mean cannabis use (range 0-8).

### Treatment Apps

#### CBT-I Coach

**Figure 1:**
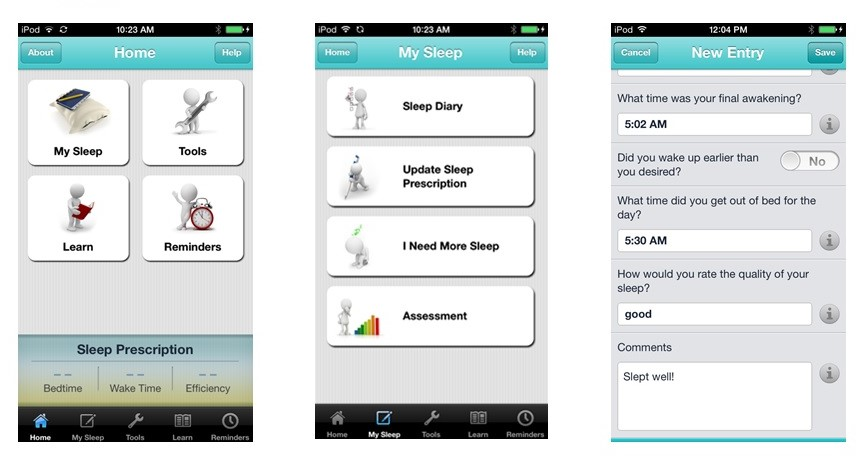
Example screenshots from CBT-i Coach.

Cognitive behavioral therapy for insomnia (CBT-I) was administered via the CBT-I Coach mobile app for iOS [39]. The content of CBT-I coach mirrors that provided by traditional CBT-I and includes four main interactive content areas: “My Sleep”, “Tools”, “Learn”, and “Reminders”. “My Sleep” provides individualized tracking of sleep and sleep prescriptions for sleep restriction (see Figure 1). Here, individuals can complete sleep diaries, update and track sleep prescriptions, complete sleep assessments to determine patterns in sleep disruption, and obtain individualized suggestions for improving sleep. Guidelines for sleep restriction and determining the prescribed sleep and rise times were completed during the baseline session with study staff. Sleep and rise times were then entered into the CBT-I Coach, which created alarms and reminders as well as tracking wake and rise times for compliance to the sleep restriction prescription. The “Tools” section provides (1) psycho-education consistent with CBT-I strategies and framework including sleep hygiene tips and stimulus control instructions, (2) guided relaxation tools that can be used in the moment to assist in treatment outcomes (eg, guided relaxation, guided worry time, cognitive restructuring exercises), and (3) individualized recommendations for relapse prevention. The “Reminders” section includes menus to customize and set reminders to (1) complete assessments, (2) set alarms for prescription bed and rise times, and (3) begin wind-down time. Finally, the “Learn” section includes psycho-education on stages of sleep, why we sleep, additional sleep disorders, and treatment including CBT-I. A glossary of terms is also provided as well as recommended good sleep habits. Participants randomized to this condition were provided with an iPod touch with CBT-I coach for the duration of the study.

#### Mood-Tracking App

A self-monitoring mobile app [40] was provided to those randomized to the control condition to test the feasibility of implementing a placebo mobile app. The iOS-based mood-tracking app is a self-monitoring tool, which allows for tracking and referencing emotional experiences (ie, sadness, happiness, stress, anxiety) over time using a visual analogue rating scale. The design of the mobile app is similar to that of CBT-I coach, and symptom rating scales are comparable across apps. Participants randomized to this condition were provided with an iPod touch with the mood-tracking app for the duration of the study.

### Procedure

Participants were recruited from a VA hospital in the San Francisco Bay Area via flyers placed throughout the hospital campus and by referrals from clinical staff. Recruitment materials were designed to target veterans interested in making a cannabis cessation attempt. Interested individuals contacted the research lab and completed a brief phone screen to assess for initial eligibility. Those meeting initial eligibility criteria were scheduled for a baseline appointment 1 day prior to the date that they would be willing to make an independent, self-guided, cannabis cessation attempt.

On arrival for the baseline appointment, individuals provided written informed consent. Individuals consented to participate in a cannabis cessation attempt and to engage with a behavioral program via mobile app. The inclusion of a sleep intervention was not discussed in recruitment or consenting procedures. Following informed consent, participants were interviewed with the SCID I-N/P and then completed a battery of self-report measures. Following the completion of the baseline assessment, participants were randomly assigned, by coin flip, to receive either the CBT-I (experimental condition) or mood-tracking app (control condition). Following randomization, participants were then loaned an iPod touch with charger, trained on how to use their assigned mobile app (which was pre-installed), and instructed to (1) make a cannabis cessation attempt beginning the following morning, and (2) use their assigned mobile app during the first 2 weeks of their cessation attempt. At the end of the baseline assessment, contact information was obtained for each participant to allow for reminder calls regarding app use and follow-up appointment date. Participants were then scheduled for their 2-week follow-up and compensated US $45 for completion of the baseline appointment.

Two weeks following baseline, participants returned to the research laboratory to complete a self-report assessment battery that was similar to the baseline assessment, with the exception of the SCID I-N/P and addition of the MAUM. Following the 2-week follow-up assessment, participants were instructed to relinquish their iPod touch devices and were compensated US $45. All study procedures were approved by the VA Palo Alto and Stanford Institutional Review Boards.

### Data Analytic Plan

Based on the small sample size of this pilot study, study outcomes (cannabis use, sleep, and metrics of mobile phone app use) were examined qualitatively for each participant separately.

## Results

Information regarding baseline and follow-up scores on study variables can be found in Table 1. Below is an overview of cannabis use and sleep for each participant during baseline and follow-up assessments.

### Individual Results

#### Participant 1

**Figure 2:**
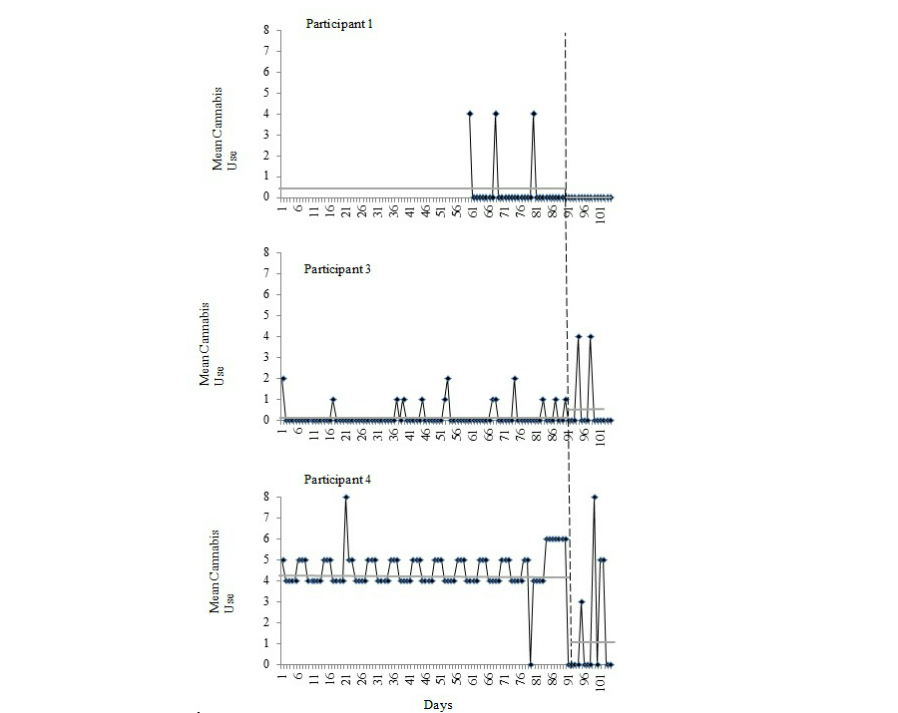
Quantity of cannabis use during each day of the 90-day (30-days for participant 1) baseline and 2-week follow-up periods for each participant, as measured by the MSHQ (Participant 1) and TLFB (Participants 2-4). Vertical dashed line indicates the cessation day for each participant. Because Participant 2 did not complete the follow-up assessment, his data are not presented. Horizontal gray solid lines represent mean cannabis use during the represented timeframe (baseline, 2-week follow-up).

Participant 1 was a 42-year-old single Caucasian male veteran who met DSM-5 criteria for current cannabis use disorder and alcohol use disorder. Participant 1 was a non-smoker and did not meet criteria for any mood or anxiety disorders. He was a novice with mobile technology and did not own a smartphone. As noted in the Method section, the MSHQ was administered instead of the TLFB for this participant. Based on the number of reported days using cannabis during the prior month, coupled with the quantity reported, we were able to extrapolate this participant’s cannabis use during the 30 days prior to baseline (see Figure 2). From this, we were able to determine that the participant’s mean cannabis use for the 30 days preceding his baseline appointment was 0.40 (on the 0-8 visual scale [36]). In terms of baseline sleep, a total PSQI score of 9 was observed, with a notable impairment in sleep efficiency (ratio of time asleep to time spent in bed trying to sleep; 58%). He reported no use of medications (prescription or over-the-counter) for sleep.

Participant 1 was randomly assigned to receive CBT-I coach. He reported using the mobile app daily over the 2-week course of his cessation attempt. On average, Participant 1 used the app for 5 minutes at a time and typically interacted with the app in the morning. He reported the most helpful aspect of the app as allowing him to track and view his sleep over the 2-week period. He reported the app as user-friendly and indicated interest in using it in the future. Participant 1 also reported that the app helped his sleep because it provided daily accountability to the intervention components. He also noted that it helped with the cessation attempt because it made him more aware of when and how much cannabis he was using. At 2-week follow-up, Participant 1 had self-reported no cannabis use during the 2-week study period. His total PSQI score did not change from baseline (total score=9); however, his sleep efficiency increased from 58% to 80%, bringing it to just below the normal range (85%). At follow-up he reported using medication (prescription or non-prescription) less than once a week for sleep.

#### Participant 2

Participant 2 was a 54-year-old widowed black/non-Hispanic male veteran who met DSM-5 criteria for current cannabis use disorder and alcohol use disorder. He did not meet criteria for any mood or anxiety disorders. Participant 2 was a tobacco smoker, consuming an average of 4 cigarettes per day. His mean cannabis use over the 90 days prior to the baseline appointment was 0.71 (on the 0-8 visual scale [36]) and his baseline PSQI total score was 19, with a sleep efficiency of 28%, which indicates a clinically significant level of sleep disturbance [31]. He reported using medication (prescription or non-prescription) once or twice a week for sleep.

Participant 2 was randomly assigned to the control condition (mood-tracking app). This participant dropped out of the study (ie, did not complete his 2-week appointment), thus follow-up data are not available.

#### Participant 3

Participant 3 was a 65-year-old married black/Hispanic male veteran who met DSM-5 criteria for cannabis use disorder. He did not meet criteria for any mood or anxiety disorders. Participant 3 was also a novice with mobile apps and did not own a smartphone. His mean cannabis use over the 90 days prior to the baseline appointment was 0.18 (on the 0-8 visual scale [36]; see Figure 1). The participant’s sleep at baseline was consistent with significant sleep disturbance (PSQI total score=17 [31]) and impaired sleep efficiency (29%). He reported using medication (prescription or non-prescription) less than once a week for sleep.

Participant 3 was randomized to the control condition. He reported using the mood-tracking app minimally (less than once per week) over the 2-week cessation period. Participant 3 reported the app as extremely user-friendly, though he indicated that he would not use it in the future and felt that it did not help at all with his cannabis cessation attempt or sleep. On follow-up, Participant 3 reported an increase in cannabis use (mean use 0.57; on the 0-8 visual scale [36]), as compared to baseline. A slight improvement in sleep was observed (PSQI total score=14), though overall sleep remained indicative of clinically significant levels of sleep disturbance. Sleep efficiency also improved, with his self-reported sleep efficiency score increasing from 29% to 41% at follow-up. He also reported that he did not use medication (prescription or non-prescription) for sleep.

#### Participant 4

Participant 4 was a 27-year-old divorced Caucasian male veteran of recent conflicts in Iraq and Afghanistan. SCID I-N/P assessment indicated that he met criteria for current PTSD and cannabis use disorder; he was a tobacco non-smoker. Participant 4 had the most experience (of the study participants) with mobile technology, and owned an Android smartphone. He reported the highest amount of cannabis use in the 90 days prior to baseline relative to other participants, with a mean use of 4.51 (on the 0-8 visual scale [36]; see Figure 1). Participant 4’s baseline sleep met criteria for mild sleep disturbance (PSQI total=5), with a reported sleep efficiency of 84%. He did not use medication (prescription or non-prescription) for sleep.

Participant 4 was randomly assigned to the CBT-I coach app. He reported using the app daily over the course of the 2-week intervention period. On average, he used the app for 10 minutes during each use, typically in the morning. He noted the app as very user-friendly and indicated that he would use it again in the future. Participant 4 reported that the stimulus control and sleep prescription components of the app were the most helpful, highlighting that the app was useful for sleep, but not for his cannabis cessation attempt. On follow-up, a decrease in mean cannabis use was observed during weeks 1 and 2 (mean use 1.50 on the 0-8 visual scale [36]; see Figure 1). The participant also reported improvement in sleep, with an observed follow-up PSQI total score of 2 and a sleep efficiency increasing from 80% to 100%. In addition, he did not use any medications (prescription or non-prescription) for sleep at follow-up.

**Table 1:** Participant-level cannabis use and sleep data for baseline and follow-up assessments.

Variable		P1: Experimental		P2: Placebo control		P3: Placebo control		P4: Experimental	
Time of assessment		Baseline	Follow-up	Baseline	Follow-up	Baseline	Follow-up	Baseline	Follow-up
Cannabis use^a^		0.40	0.00	0.71	N/A	0.18	0.57	4.51	1.50
**Sleep** ^b^									
	Quality	1	1	3	N/A	3	3	1	1
	Latency	30 min	10 min	30 min	N/A	120 min	120 min	30 min	15 min
	Duration	5 hours	6 hours	2 hours	N/A	2.5 hours	3.5 hours	8 hours	8 hours
	Efficiency	58%	80%	28%	N/A	29%	41%	80%	100%
	Disturbances	2	2	3	N/A	3	2	1	0
	Med use	0	1	2	N/A	1	0	0	0
Daytime dysfunction		0	2	3	N/A	1	0	1	1
Total PSQI^c^		9	9	19	N/A	17	14	5	2

^a^Mean cannabis use was based on both frequency of use (ie, number of days) and quantity of use per day (scores range from 0-8). Quantity of cannabis used on each day was indexed by a graphical depiction of joints of varying sizes that ranged from 0-8 [36].

^b^Sleep quality, sleep latency, sleep duration, sleep efficiency, sleep disturbances, daytime dysfunction, and sleep med use represent the 7 components of the PSQI [31]. Sleep quality, sleep disturbances, and daytime dysfunction range from 0 “best” to 3 “worst.” In terms of sleep medication use, 0=no use in the past month, 1=less than once a week, 2=once or twice a week, and 3=three or more times a week.

^c^PSQI total scores can range from 0 (excellent sleep) to 21 (very poor sleep).

### Feasibility

#### Overview

The current study provided an initial demonstration of using mobile apps for the delivery of psychological interventions among veterans. Feasibility was examined in terms of recruitment, costs, and veteran engagement with the mobile app.

#### Acceptability Among Veterans

Overall, participants reported interest in using the mobile apps, describing them as easy to use and accessible. Importantly, this feasibility study demonstrated reported app accessibility among veterans without experience with mobile technology. In addition, this study demonstrated the feasibility of using mobile apps among multiple age groups. Indeed, participants ranging from their mid-20s to mid-60s all reported ease in using the mobile apps.

#### Costs

Individuals were compensated up to a total of US $90 for participation. Additional research costs included iPod touch devices (only 1 individual in the study had their own iPhone) and staff costs.

#### Mobile App Use

Overall, our sample consisted of novices in terms of mobile technology and mobile app use. Though Participant 4 had experience with using mobile apps, all other participants reported not owning a smartphone or having previous experience with mobile apps. However, all were able to use the apps after a brief explanation. The responses and engagement with the mobile apps was found to vary by treatment condition. Participant 3 (control) reported minimal use of the app (scoring a 1 out of a possible 4). Follow-up assessment of app use found that this individual did not find the mood tracking app helpful for sleep (reporting 0 out of 4) or cannabis cessation (reporting 0 out of 4) and therefore reported little interest in engaging with the app and low likelihood of using it in the future (reporting 0 out of 4). However, for those who received the CBT-I app (Participants 1 and 4), daily use was reported over the 2-week study period, with an average of 5-10 minutes spent with the app per use session.

Participants assigned to the CBT-I app reported that the most helpful portions of the app were the sleep logs and the exercises for improving sleep. Both reported the app to be very user-friendly (scoring 3 out of 4), found that it was very helpful in improving sleep (scoring 3 out of 4), and reported strong interest in using an intervention app in the future (scoring 3 out of 4). All loaned iPod touches were returned to the study team after completion of the study. Although Participant 2 did not complete the 2-week follow-up, he did return to the laboratory to return his iPod touch.

## Discussion

### Principal Findings

This pilot study served as a feasibility test of mobile-app delivered CBT-I (and mood-tracking control) for both cannabis and sleep outcomes among individuals engaged in a cannabis cessation attempt. This study indicates that individuals who received the CBT-I app reported daily engagement with the app, while individuals in the control condition either dropped out of the study or reported minimal use. Importantly, regardless of condition, this study provided support for loaning out iOS devices for the purposes of treatment, as all devices were returned following study completion.

Findings support the initial feasibility of providing CBT-I via mobile app to improve outcomes among individuals engaged in a cannabis cessation attempt. Coupled with theoretical and empirical work that has highlighted the pivotal role of sleep in the maintenance and treatment of cannabis use disorders [8], including among vulnerable populations (eg, those with posttraumatic stress disorder [41]), these data indicate the clinical importance of continuing to comprehensively understand the efficacy and effectiveness of CBT-I for cannabis cessation. Indeed, despite its widespread dissemination [42], CBT-I is not currently indicated or utilized as a treatment for substance use disorders, particularly within the Veterans Health Administration. Although these findings are preliminary, this study highlights the potential utility and feasibility of providing CBT-I for patients with a cannabis use disorder. Further, the observation that CBT-I can be effectively implemented via mobile app on loaned devices addresses some of the barriers to care among this population, particularly access and stigma [24-27] and provides additional evidence for the growing movement toward technology-based intervention delivery [28].

### Limitations

Although our investigation served as a novel extension of prior empirical and theoretical work, it was not without limitations. First, the small number of participants, all of whom were male, enrolled in this preliminary study limits inference regarding generalizability of findings. Future work would benefit from the replication and extension of this pilot study to a larger and more gender diverse sample. Future work would benefit from focusing recruitment efforts to target female participants. Second, while we screened 40 individuals, due to our inclusion criteria, only four were enrolled. This may have biased the randomness of our sample. Third, given the scope of the study, follow-up data were collected only 2-weeks post-baseline. As the impact of cannabis cessation on sleep can last for over 30-days post-cessation [15], future work should assess patients within a longer follow-up period to determine whether the impact of CBT-I on sleep and cannabis outcomes continues to improve, or begins to abate, as time progresses. Fourth, study participants with the worst baseline sleep problems were randomized to the control condition, a function of the small sample size. Thus, at this point, the efficacy of the CBT-I app for improving severe sleep problems among treatment-seeking cannabis users remains to be determined. Fifth, we examined CBT-I delivery via mobile app and did not include a therapist delivery condition. Future research should compare both methods of delivery as well as including cost-effectiveness analyses. Sixth, while individuals were randomized to groups, we did not have an appropriate sample size to conduct group-based analyses. Future work should include age and other relevant demographic variables as covariates in analyses. Finally, though many of the measures employed for the assessment of cannabis and sleep are considered “gold standard” self-report instruments, the addition of multi-method assessments in future work, including objective measures of cannabis use (eg, plasma, urinalysis) and sleep (eg, actigraphy, polysomnography), would strengthen confidence in self-reported behaviors.

### Conclusions

This study provides initial evidence for the feasibility of CBT-I for cannabis dependence delivered through a mobile phone app. Findings support the use of technology to enable easy, affordable access to treatment for substance use disorders [43-45], and small sample studies to address the challenges in testing new technology-based interventions [46]. CBT-I shows promise to treat cannabis use disorder and associated sleep difficulties. Its efficacy should be evaluated in a randomized controlled trial powered to detect treatment effects on cannabis use and sleep.

## Abbreviations

CBT-I: Cognitive Behavioral Therapy for Insomnia
DSM-IV: Diagnostic and Statistical Manual of Mental Disorders, 4th edition
DSM-5: Diagnostic and Statistical Manual of Mental Disorders, 5th edition
MAUM: Mobile App Use Measure
MSHQ: Marijuana Smoking History Questionnaire
PSQI: Pittsburgh Sleep Quality Index
PTSD: posttraumatic stress disorder
SCID I-N/P: Structured Clinical Interview–Non-Patient Version for DSM-IV
TLFB: Timeline Follow-Back Interview
